# Supplementary figures and images for: 1,5-Anhydro-D-Fructose Exhibits Satiety Effects via the Activation of Oxytocin Neurons in the Paraventricular Nucleus
Source: Int J Mol Sci. 2023 May 4;24(9):8248. doi: 10.3390/ijms24098248 (PMC10179633; doi:10.3390/ijms24098248)

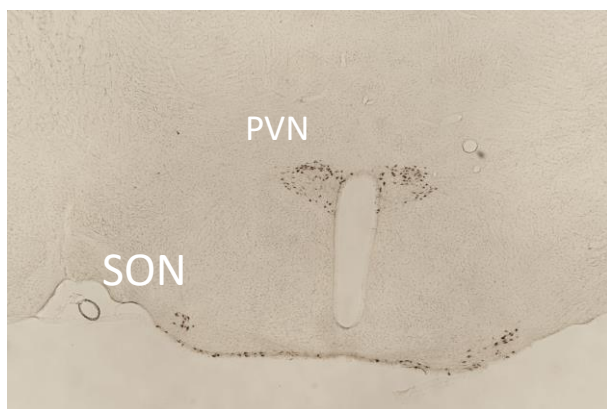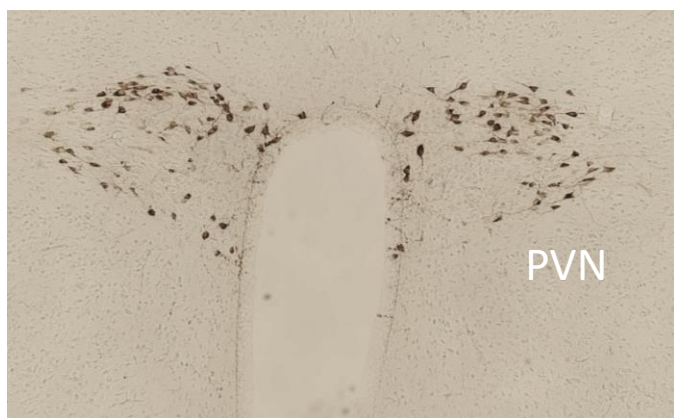

**Supplementary Figure S1. Immunostaining using anti-oxytocin monoclonal antibody**

Supplement: Supplementary file 1 [file ijms-24-08248-s001.zip › ijms-2338150-supplementary.pdf]
